# Premature synaptic mitochondrial dysfunction in the hippocampus during aging contributes to memory loss

**DOI:** 10.1016/j.redox.2020.101558

**Published:** 2020-05-05

**Authors:** Margrethe A. Olesen, Angie K. Torres, Claudia Jara, Michael P. Murphy, Cheril Tapia-Rojas

**Affiliations:** a Laboratory of Neurobiology of Aging, Centro de Biología Celular y Biomedicina (CEBICEM), Universidad San Sebastián, Chile; b Medical Research Council Mitochondrial Biology Unit, University of Cambridge, Cambridge Biomedical Campus, Cambridge, UK

**Keywords:** S**ynaptic**, **Non-synaptic**, **Mitochondria**, **Aging**, **Hippocampus**, **Memory**

## Abstract

Aging is a process characterized by cognitive impairment and mitochondrial dysfunction. In neurons, these organelles are classified as synaptic and non-synaptic mitochondria depending on their localization. Interestingly, synaptic mitochondria from the cerebral cortex accumulate more damage and are more sensitive to swelling than non-synaptic mitochondria. The hippocampus is fundamental for learning and memory, synaptic processes with high energy demand. However, it is unknown if functional differences are found in synaptic and non-synaptic hippocampal mitochondria; and whether this could contribute to memory loss during aging. In this study, we used 3, 6, 12 and 18 month-old (mo) mice to evaluate hippocampal memory and the function of both synaptic and non-synaptic mitochondria. Our results indicate that recognition memory is impaired from 12mo, whereas spatial memory is impaired at 18mo. This was accompanied by a differential function of synaptic and non-synaptic mitochondria. Interestingly, we observed premature dysfunction of synaptic mitochondria at 12mo, indicated by increased ROS generation, reduced ATP production and higher sensitivity to calcium overload, an effect that is not observed in non-synaptic mitochondria. In addition, at 18mo both mitochondrial populations showed bioenergetic defects, but synaptic mitochondria were prone to swelling than non-synaptic mitochondria. Finally, we treated 2, 11, and 17mo mice with MitoQ or Curcumin (*Cc*) for 5 weeks, to determine if the prevention of synaptic mitochondrial dysfunction could attenuate memory loss. Our results indicate that reducing synaptic mitochondrial dysfunction is sufficient to decrease age-associated cognitive impairment. In conclusion, our results indicate that age-related alterations in ATP produced by synaptic mitochondria are correlated with decreases in spatial and object recognition memory and propose that the maintenance of functional synaptic mitochondria is critical to prevent memory loss during aging.

## Highlights

• Hippocampus-dependent learning and memory are impaired with age, which correlated with synaptic mitochondrial dysfunction.
• Synaptic mitochondria fail before non-synaptic mitochondria, indicating premature synaptic mitochondrial damage in aging.
• Reducing synaptic mitochondrial dysfunction, with MitoQ or Curcumin, decrease age-associated hippocampal memory impairment.
• Age-related changes in ATP production of synaptic mitochondria correlated with decreased hippocampal memory.
• Maintenance of functional synaptic mitochondria is critical to prevent memory loss during aging.

## Graphical abstract

**Figure:**
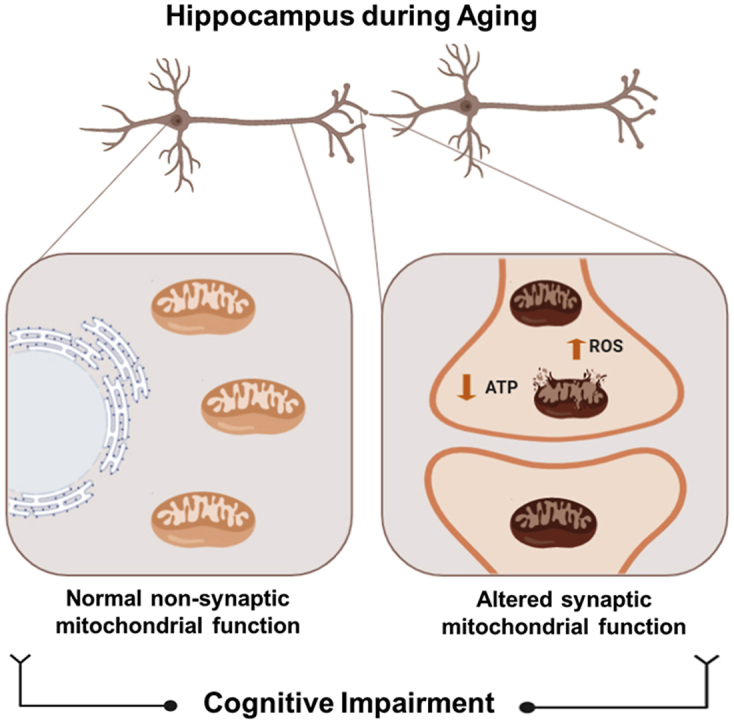

## 1 Introduction

Aging is a multifactorial process, characterized by deterioration of physiological and cellular functions [1], including brain function [2]. One of the most affected functions is memory, requiring more time to carry out the learning and memory process [3]. The hippocampus plays an important role in memory [4]; storing information associated with the recognition of an event (recognition memory), as well as spatiotemporal context (spatial memory) [5]. However, hippocampal atrophy is observed in aging, which could explain the age-associated memory deficit [6,7].

Studies have shown the importance of mitochondria in synaptic communication as well as to hippocampus-dependent learning and memory [8]. Mitochondria supply energy, maintain calcium homeostasis and regulate the redox balance [9]. The internal mitochondrial membrane contains the electron transport chain (ETC) that generates ATP [10] and as a secondary product form reactive oxygen species (ROS) [11]. Oxidative molecules act as cellular regulators [12]; however, its overproduction generates oxidative stress, which is strongly associated with aging [[13], [14], [15]]. In addition, mitochondrial calcium regulation is mediated by transient mitochondrial permeability transition pore (mPTP) opening [16]. Nevertheless, in conditions of high mitochondrial calcium, the mPTP opening is induced, generating mitochondrial swelling and apoptosis [17]. During the last few decades, it has been suggested that mitochondrial dysfunction plays an important role in aging [18]. Aged mitochondria are incapable of regulating calcium; they present decreased ATP production, and increased ROS generation; which result in bioenergetic defects and oxidative damage [14,19,20]. In addition, mitochondrial dysfunction is considered a hallmark of aging [14,21] and could contribute to the loss of cognitive abilities observed with age [14,22].

In the brain, mitochondria have been classified into non-synaptic and synaptic mitochondria [23]. Non-synaptic mitochondria come from neuronal and glial cells, whereas synaptic mitochondria are exclusively found in neurons, specifically in the synapses [24]. Pre-synaptic mitochondria are necessary to produce ATP required for the release of neurotransmitters [25]; whereas post-synaptic mitochondria are fundamental to the synaptic transmission [26]. Increasing evidence suggests that synaptic mitochondrial impairment is strongly associated with neuronal failure in Alzheimer's Disease (AD) [27]. In AD, synaptic mitochondria show increased ROS production, decreased respiration rate, and impaired calcium regulation; which occur before the alterations in non-synaptic mitochondria and the appearance of the AD pathology [27]. Interestingly, synaptic mitochondria from the cerebral cortex of 3month-old (mo) rats are more susceptible to high calcium concentrations than non-synaptic mitochondria [28] and fail earlier than non-synaptic mitochondria at advanced age [29,30]. Considering this evidence and the importance of the hippocampus to learning and memory, we proposed that hippocampal synaptic mitochondria failure could occur before non-synaptic mitochondria during aging, contributing to age-associated cognitive impairment.

Here, we studied the function of hippocampal synaptic and non-synaptic mitochondria from 3, 6, 12 and 18mo mice, and its contribution to hippocampus-dependent memory loss. We observed that 12mo mice present recognition memory impairment, while the loss of spatial memory was observed at 18mo. Interestingly, regarding mitochondrial function, we observed reduced ATP production only in the synaptic mitochondria of 12mo mice; whereas 18mo mice showed bioenergetic defects in both populations. Similarly, calcium sensibility was higher in synaptic mitochondria from 12 and 18mo mice than non-synaptic mitochondria, indicating that synaptic mitochondria fail in a premature manner compared with non-synaptic mitochondria. In addition, to validate that synaptic mitochondrial dysfunction contributes to memory impairment, 2, 11, and 17mo mice were treated with the mitochondria-targeted antioxidant MitoQ, or Curcumin (*Cc*) for 5 weeks. MitoQ consists of a ubiquinone moiety linked to a triphenyl-phosphonium moiety by a 10-carbon alkyl chain [31,32]; which improves behavior in mice after brain damage [33,34] and in a mouse model of AD [35]. Additionally, we studied the beneficial effects of *Cc*, because has been described as an anti-inflammatory and antioxidant molecule, improving inflammatory and neurodegenerative diseases [36,37]. Interestingly, we observed that treatment was sufficient to ameliorate the cognitive impairment, exclusively improving synaptic mitochondrial function. In fact, we observed a correlation between the concentrations of ATP produced by synaptic mitochondria and the cognitive performance in the Novel Object Recognition (NOR) and Morris Water Maze (MWM) tests. In conclusion, synaptic mitochondrial dysfunction occurs before that non-synaptic fail and contributes to memory loss during aging; therefore, molecules that preserve synaptic mitochondrial function could be used to prevent the development of age-associated diseases.

## 2 Materials and methods

**Reagents**: Isolation Medium Buffer (225 mM sucrose, 75 mM mannitol, 1 mM EGTA, 5 mM HEPES, pH 7.4). Percoll (GE LIFESCIENCES 17-5445-02), Bovine Serum Albumin (1120180100, Merck Millipore), respiration Buffer (125 mM KCl; 0.1% BSA; 20 mM HEPES; 2 mM MgCl2; 2.5 mM KH2PO4), Pyruvate (P2256, Sigma Aldrich), BCA Protein Assay Kit (23227, Thermo Fisher Scientific), Malate (M6413, Sigma Aldrich), CM-H2DCFDA (C6827, Thermo Fisher Scientific), ATP determination kit (A22066, Invitrogen), CaCl2 (7521789, Merck), Curcumin (C7727, Sigma Aldrich), MitoQ (Mitoquinol-Mesylate, 01ATP04C-02-13, MitoQ Ltd).

**Animals**: C57BL/6 mice male and female from 3, 6, 12 and 18mo were handled according to the guidelines of the National Institute of Health (NIH, Baltimore, MD). Animals were housed in cages at controlled temperature (24 °C), in a 12-h light/dark cycle with food and water ad-libitum. Experimental procedures were approved by the Bioethical and Biosafety Committee of the University San Sebastian, Chile. After the behavior test, the animals were anesthetized with isoflurane and killed by decapitation. Then, the hippocampus was removed for biochemical analysis. For the first part of this study, to determine the age-related cognitive and mitochondrial differences, each group was formed by an n = 8 animals. In the second part of the study, the control, MitoQ and Curcumin groups were formed by n = 6 different animals to perform the cognitive and biochemical assays.

**Mice treatment**: Mice of 3, 12 and 18mo were subjected to MitoQ or Curcumin treatment by 5 weeks. Control group was injected with saline solution and controlled water volume. Curcumin was injected intraperitoneally (25 mg/kg) 3-times/week. MitoQ was administrated in a 250 μM water solution. These doses were used because the oral administration of 250 μM MitoQ in drinking water has been demonstrated to be safe, tolerable and beneficial to aged mice, without secondary effects, after 4 weeks of administration [38]; whereas i.p. injection of Curcumin (*Cc*) is one of the most common methods of administration in mice for several weeks, where 25 mg/Kg showed positive results in diverse mouse pathological models [39,40]. MitoQ drinking water was measured (Supplementary Figure 2). The MitoQ consumption was 1.520 ± 0.3598, 1.787 ± 0.3891 and 1.743 ± 0.6061 mol/MitoQ/day/mouse by the group of 3, 12 and 18mo respectively. We not registered the body weight during both treatments, due to no apparent differences were observed with a naked eye. This observation is consistent with previous reports that indicate that neither MitoQ nor Curcumin generates changes in body mass and weight [[41], [42], [43], [44]].

**Behavioral test**: All behavioral tests were monitored by Any-MAZE Behavioral software (Stoelting Co), using the chambers and instruments manufactured or recommended by the manufacturer. All behavioral tests were performed in the 12 h light phase of the animals light/dark cycle.

**Novel object localization (NOL) test**: NOL test was performed in a 40 × 40 × 32 cm box [45], chamber provided by Stoelting Co. The software register both the head and the body of the animal. The animals were exposed to a habituation phase without objects for one day. The next day, for testing each animal was exposed to 2 identical objects for 10 min. 2 h later, the animal was exposed to an old and a new object localization. Recognition index was calculated dividing the time that the animals spend exploring the new localization by the time exploring both localizations. After each test, the box chamber was cleaned with ethanol previous to a different mouse is tested.

**Novel object recognition (NOR) test**: 2 h later NOL test, the animals were exposed to an old and a new object. Recognition index was calculated dividing the time that the animals spend exploring the new object by the time exploring both objects. After each test, the box chamber was cleaned with ethanol previous to a different mouse is tested.

**Barnes Maze (BM) test**: The mice were accustomed to a circular platform containing 20 holes where one of them is the escape chamber [46]. Four visual signals were placed around the platform. The mice were exposed to a habituation phase followed by 2 days of training, in presence of white noise. The animals learn the location of a dark escape chamber under the platform. 48 h after, the time to find the escape chamber was evaluated. After each test, the chamber was cleaned with ethanol previous to a different mouse is tested.

**Morris Water Maze (MWM) test**: The MWM task was performed as previously described [47]. The mice were trained in a circular pool (24 °C). Each animal was trained for the location of the platform. Test was performed for 10 consecutive days, with 3 trials per day, with exception of the days 6 and 7 (training off). A submerged 9 cm platform was used, with a maximum trial duration of 60 s, where each mouse was introduced in the pool from the opposite quadrant of the platform. The test was performed with 3 trials per day and the escape latency was measured. 24 h after training, the platform was removed, and we evaluate the time in which each animal remained in the platform area for 1 min.

**Extraction of an enriched fraction of hippocampal synaptosomes (containing synaptic mitochondria) and non-synaptic mitochondria**. Mitochondrial populations were obtained using a Percoll gradient [28]. The hippocampus (both hemispheres) was homogenized in Isolation Medium and centrifuged at 1300 g for 3 min (4 °C). The pellet was homogenized in Isolation Medium and centrifuged at 1300 g for 3 min (4 °C). The supernatants were centrifuged at 21200 g for 10 min (4 °C). To separate synaptosomes and non-synaptic mitochondria, a Percoll gradient was used (15%–24% - 40%) and centrifuged at 30700 g for 8 min (4 °C). Synaptosomes (containing synaptic mitochondria) were obtained between the 15% and 24% phase, while non-synaptic mitochondria between 24% and 40% phase of the gradient. Both fractions were suspended in Isolation Medium and centrifuged at 16700 g for 10 min (4 °C). BSA (10 mg/ml) in isolation Medium was added to the pellet and was centrifuged at 6900 g for 10 min (4 °C). Finally, the mitochondria were suspended in Respiration Buffer.

**Measurement of mitochondrial ROS**: ROS production was measured using 25 μM DCF (485 nm, 530 nm) [48], in the Biotek Synergy HT plate reader. 25 μg of mitochondrial protein were added to respiration buffer containing pyruvate (5 mM) and malate (2.5 mM) and incubated at 37 °C for 30 min. The maximum fluorescence of each sample minus the blank sample (in the absence of mitochondrial proteins) was analyzed.

**Measurement of ATP concentration**: ATP was measured in the supernatant of 25 μg of mitochondria after incubation with oxidative substrates, using an ATP bioluminescence assay kit, as previously described [49].

**Measurement of the calcium response**: The mitochondrial response to calcium was measured by absorbance to 540 nm (30 °C) [50] during 3 min (basal), then we added 20 μM CaCl2 and evaluated the response during 15 min. Finally, we added 200 μM CaCl2 and measured for 15 min to evaluate mitochondrial swelling.

**Transmission Electron Microscopy (TEM)**. Hippocampal samples were used according to standard procedures of the Electron Microscope Facility of the Faculty of Biological Sciences, Pontificia Universidad Católica de Chile, Santiago, Chile. For the analysis of mitochondrial membrane integrity, we consider one intact mitochondria when these mitochondria present an intact double-membrane across their whole perimeter. We count the number of total intact mitochondria per each image obtained (26,1 μm^2^), in a total of 35 images per each experimental group, and then we graph the mean ± standard error.

**Statistical Analysis**. The data were presented as graphs indicating the mean ± standard deviation. Statistical significance was determined using one-way ANOVA with Bonferroni's post-test. p-values ≤ 0.05 were considered statistically significant. In the figures, p-values between 0.01 and 0.05 are marked with one significance mark (* or #), p-values between 0.001 and 0.01 with two significance markers (** or ##) and p-values less than 0.001 are shown with three significance markers (*** or ###). * indicates significant differences with the 3mo control group. # indicates significant differences between control and treated-mice of the same age. All statistical analyses were performed using Prism software (GraphPad Software, Inc.). Pearson's correlation analysis was used to examine the relationship between ATP or ROS produced by synaptic mitochondria and recognition index of NOR test or escape latency of the Morris Water Maze in the day 10.

## 3 Results

### 3.1 Impairment of object recognition memory occurs before object localization memory

**Fig. 1:**
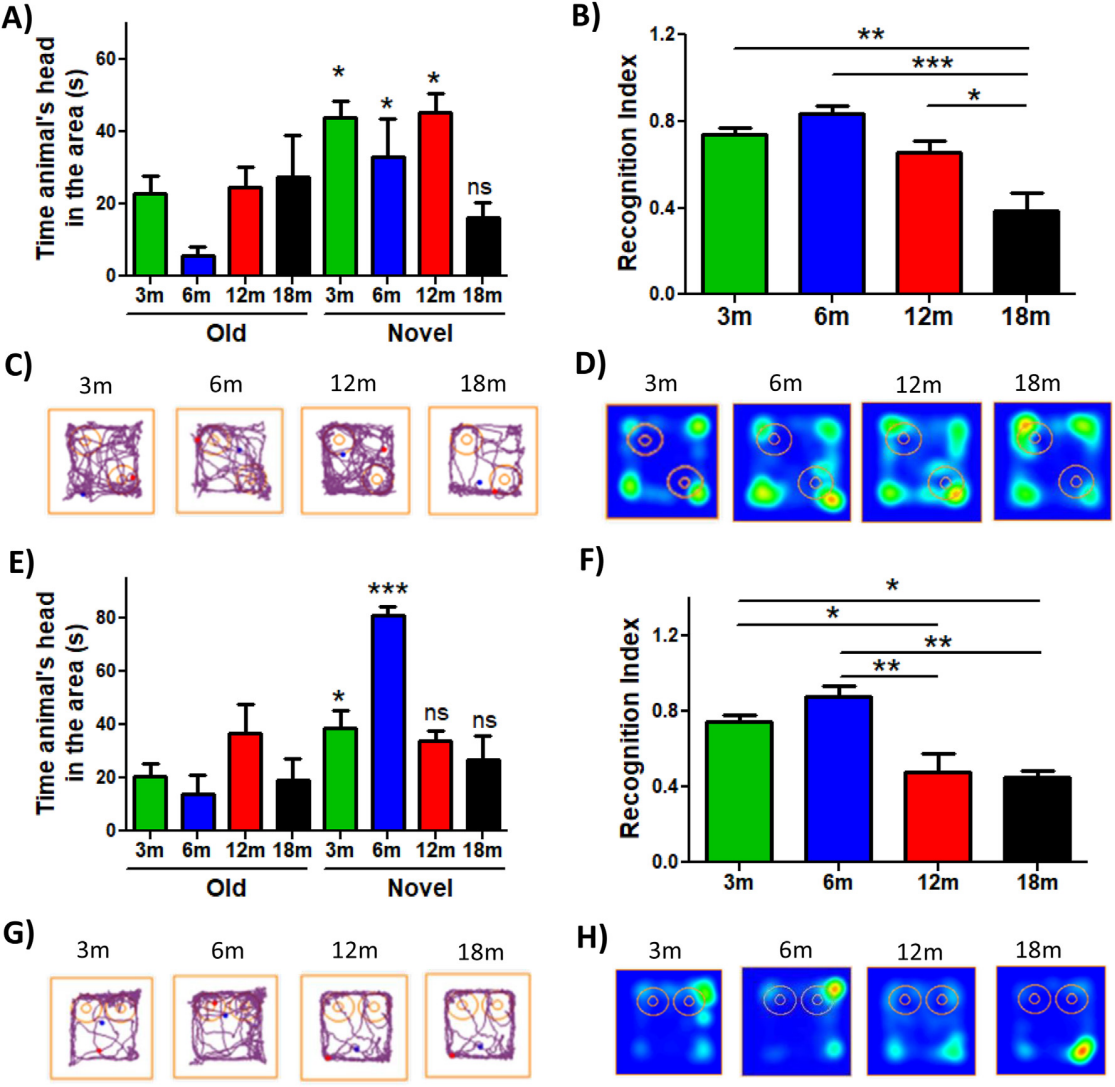
**Object localization memory and object recognition memory are differentially impaired during aging**. (A) Time that the animals explore old and novel localization of the object. (B) Recognition Index of each group. (C) Representative track of one animal per group in NOL test. (D) Heat maps of each group in the NOL test. (E) Time that the animals explore old and novel objects. (F) Recognition Index of each group. (G) Representative track of one animal from the group in the NOR test. (H) Heat maps of each group in the NOR test. Graph bars represent means ± SEM. *p < 0.05. **p < 0.01; ***p < 0.001.

For several years, researchers have studied memory loss during aging [51]. Recognition memory is a type of hippocampus-dependent memory, specifically of the CA3 region [52], which is affected during aging [53]. Here, we evaluated changes in recognition memory with age. We performed the Novel Object Localization (NOL) and Novel Object Recognition (NOR) test (Supplementary Fig. 1A and 1B) [49] in 3, 6, 12, and 18mo C57BL/6 mice. To carry out these tests, we first exposed the animals to a habituation phase, in which each animal explored the empty chamber (without objects present) for 5 min. The next day, the mice were subject to the familiarization phase. In this stage, each animal had 10 min to explore the chamber, which contained two identical objects. After 2 h, the NOL stage was performed. In this phase, the animals explored the same objects for 5 min, but one object was localized in other position in the chamber (Supplementary Figure 1A). We observed that the 3, 6, and 12mo mice exhibited more time exploring the novel localization of the object, as indicated by the time that the animal's head spent in this area (Fig. 1A). In contrast to 18mo mice, which showed no preference by the novel localization, observing that this group spent similar time exploring both object locations (Fig. 1A). This was more evident when we analyzed the Recognition Index, which represents the time spent exploring the localization of the novel object relative to the total time exploring both localizations (Fig. 1B). We observed that 3, 6, and 12mo mice showed a higher preference for the localization of the novel object compared to 18mo mice (Fig. 1B). The differences in explorative behavior are shown in the representative traces of each group (Fig. 1C) and in the heat maps (Fig. 1D), where only 18mo mice showed no preference for the new location of the object, remaining similar time exploring both object locations. These results indicate that 18mo aged mice are incapable of recognizing the novel localization of the object, suggesting that at 18mo object localization memory is impaired.

**Fig. 2:**
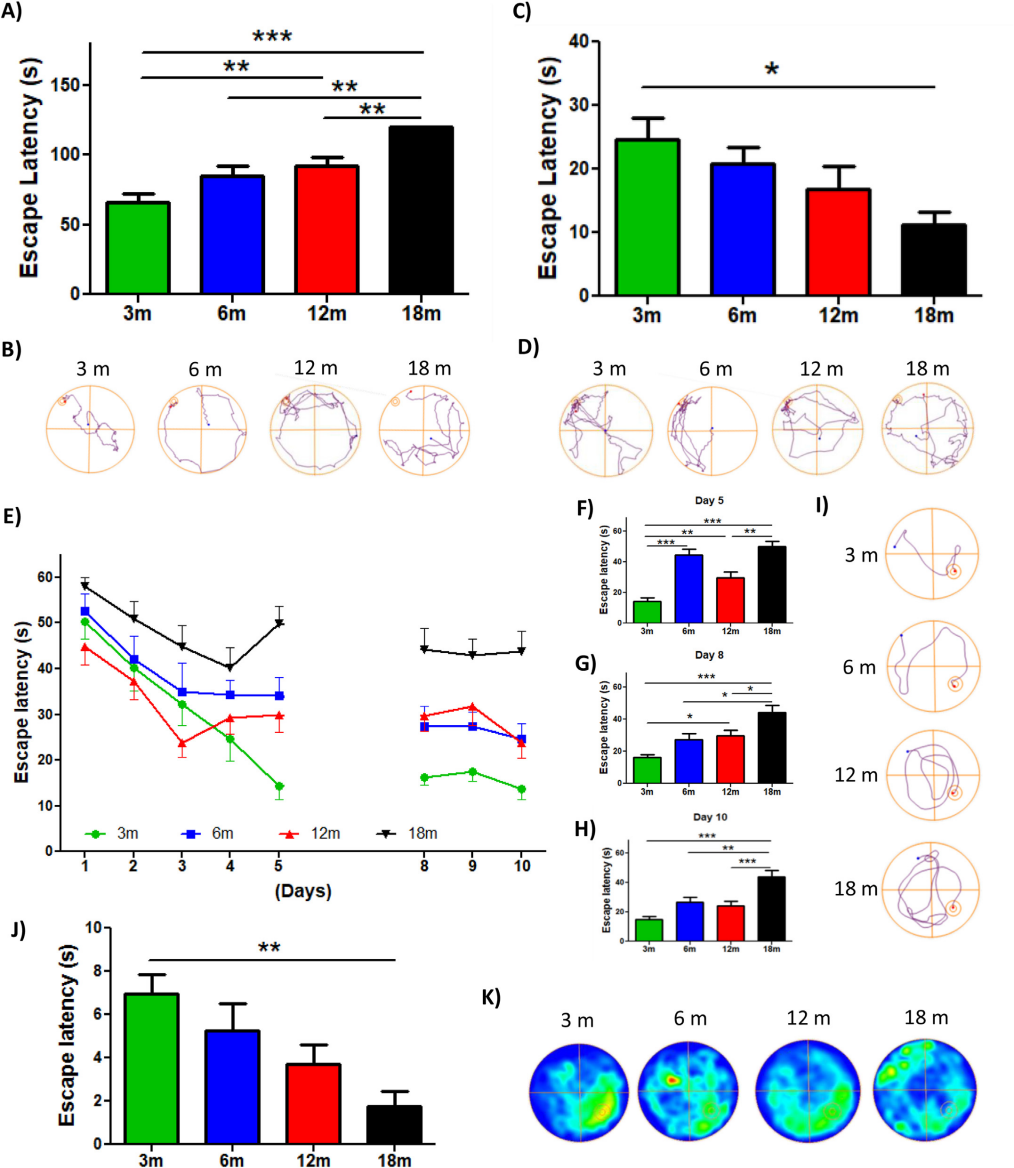
**Spatial memory loss is observed in animals of 18 month-old**. (A) Time that the animals spent to find the escape chamber during BM training. (B) Representative track of one animal per group during BM training. (C) Time that each group stayed in the escape area. (D) Representative track of one animal per group during the BM test. (E) Escape latency during the MWM test. Significant differences during the (F) 5th day, (G) 8th day and (H) tenth day. (I) Representative track of one animal per group during the 10th day of MWM. (J) Time that each group spent in the area of the platform during the Probe test. (K) Heat maps of each group in the Probe test. Graph bars represent means ± SEM. *p < 0.05. **p < 0.01; ***p < 0.001.

Diverse studies performed in rats, monkeys, and humans indicate that recognition memory is impaired at an advanced age [54]. Considering this, object recognition memory also was evaluated (NOR test). For this, 2 h after the NOL test, a familiar object was replaced by a novel object (Supplementary Figure 1B) [49]. In this phase, animals explored both old and novel objects for 5 min. We observed that 3 and 6mo mice spent more time exploring the novel object compared with 12 and 18mo mice, which spent a similar time exploring both objects (Fig. 1E). Similarly, this is observed in the Recognition Index (Fig. 1F), the representative track (Fig. 1G) and the heat map (Fig. 2H), where 12 and 18mo mice presented significantly reduced novel object recognition. Thus, these results indicate a loss of object recognition memory since 12mo in this mouse line. Altogether, our findings indicate that both object localization and recognition memory are impaired with age; however, defects in object recognition memory appear before localization memory.

### 3.2 Loss of spatial memory is observed in 18 month-old mice

The hippocampus is a crucial structure for spatial memory, associated with mental images that help to recognize characteristics of the environment [55]. For several years, researchers have shown that the loss of spatial memory is associated with aging [56]. Here, we evaluated spatial memory using the Barnes Maze (BM) (Supplementary Figure 1C) [46] and Morris Water Maze (MWM) task (Supplementary Figure 1D) [47] (Fig. 2). In the Barnes Maze test, the animals were exposed to a training phase, where animals had to find the location of a hole containing an escape chamber within 2 min (Fig. 2A and B). After 48 h of the last training session, animals had to find the location of the escape hole in the absence of the escape chamber (Fig. 2C and D). Our results showed that in the training stage, the 18mo animals took a longer time to find the escape chamber compared to 3, 6, and 12mo animals (Fig. 2A). The training track is observed in Fig. 2B. Finally, after 48 h the 18mo animals remembered the location of the escape chamber significantly less compared to other groups (Fig. 2C). This was also evident in the representative track (Fig. 2D). Therefore, in this test we observed that 3, 6, and 12mo mice learned and remember the spatial location of the escape chamber, in contrast to 18mo animals; suggesting that spatial memory is reduced with age, specifically at 18mo.

To validate this last observation, we used the MWM test, where each animal was placed 3 times per day in a pool to find the hidden escape platform guided by spatial cues, for 10 days. We observed that during the first 5 days of training 3, 6, and 12mo mice quickly learn the location of the hidden platform, in contrast to 18mo mice; nevertheless, it was also observed that the 6 and 12mo mice reduced their learning from day 3 of the MWM test (Fig. 2E). After a 48 h break, 3, 6, and 12mo mice remembered the location of the platform, meanwhile, 18mo mice had higher escape latency (Fig. 2E). Statistical analyses revealed that during the 5th day of training 3 and 12mo mice found the platform in less time than 18mo mice (Fig. 2F). Similarly, on the 8th day of training, 3, 6 and 12mo groups presented significant differences compared to 18mo mice (Fig. 2G). Also, it was possible to observe that 12mo mice spend more time to find the platform compared to the 3mo group, suggesting that the 48 h delay negatively affected the memory of 12mo animals (Fig. 2G). Interestingly, on the last day of training, all experimental groups showed significant differences compared to the 18mo group, which spent more time finding the hidden platform (Fig. 2H). Analyzing the track of the 10th day, we observed that the 3, 6 and 12mo animals showed a shorter path towards the platform than the 18mo group (Fig. 2I). Finally, on the 11th day, we performed the Probe test, which consisted of removing the platform to evaluate the time that the animals explored the platform zone. 3mo mice spent significantly more time in the platform area compared to 18mo animals (Fig. 2J). There was also a gradual reduction in the time spent exploring the platform area as age increased (Fig. 2J), which was shown by the heat maps of Fig. 2K. Thus, the MWM test also revealed an impairment in spatial memory at 18mo. Therefore, these results indicate an impairment of learning and spatial memory during aging, specifically at 18mo.

In summary, our behavior studies showed that hippocampal-dependent memory is affected with age; the object recognition memory was first impaired at 12mo; whereas the localization and spatial memory were affected at 18mo in this mouse background.

### 3.3 Synaptic mitochondrial dysfunction occurs before non-synaptic mitochondria in the hippocampus during aging

**Fig. 3:**
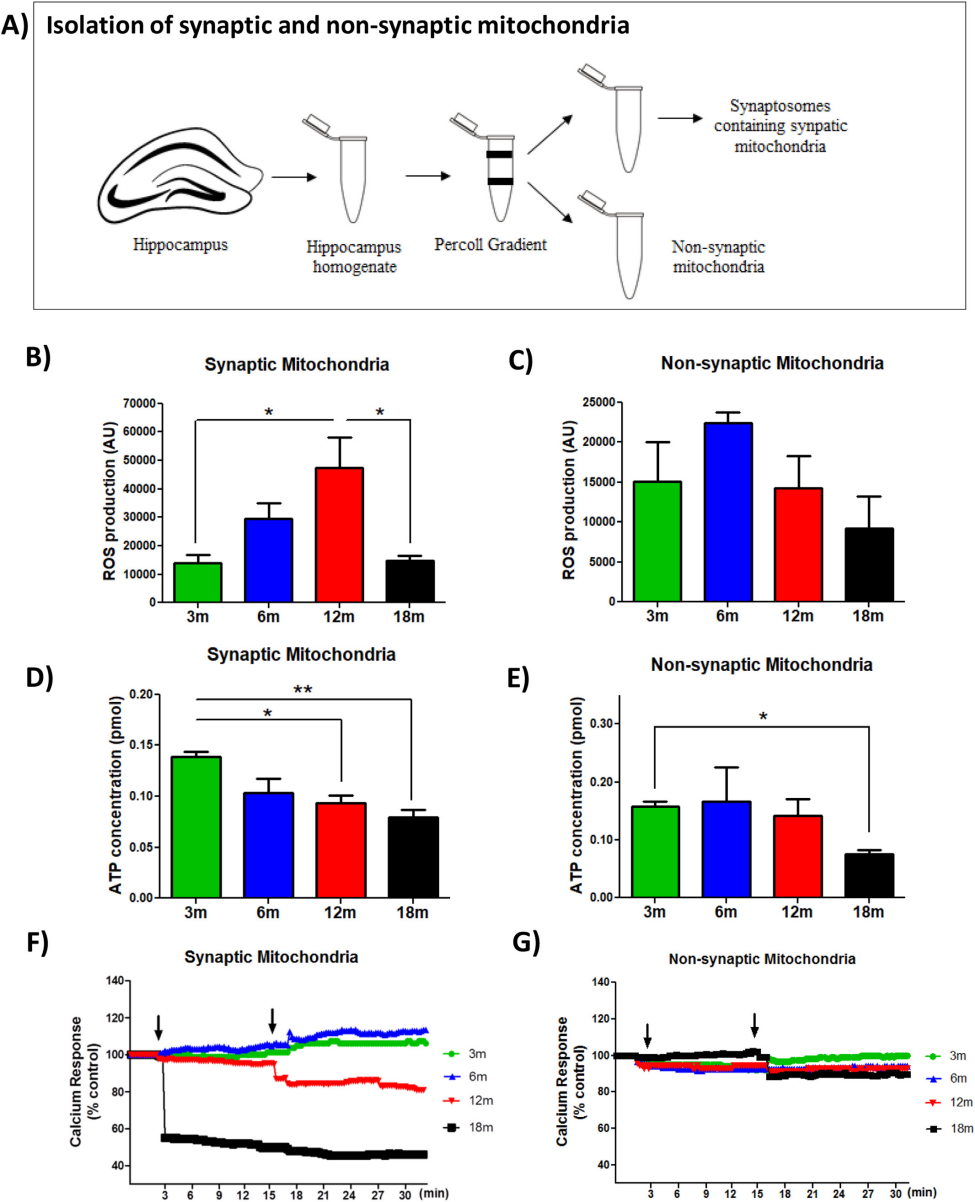
**Premature dysfunction of synaptic mitochondria compared with non-synaptic mitochondria during aging** (A) Representation of synaptic and non-synaptic mitochondrial isolation from the hippocampus through Percoll gradient. ROS production by (B) synaptic and (C) non-synaptic mitochondria 30min after exposure to oxidative substrates. ATP concentration produced by (D) synaptic and (E) non-synaptic mitochondria 30min after exposure to oxidative substrates. Response to calcium overload by (F) synaptic and (G) non-synaptic mitochondria after exposure to 20 μM and 200 μM CaCl_2_. Graph bars represent means ± SEM. *p < 0.05. **p < 0.01.

Due to the high energetic demand, mitochondria are fundamental for the functioning of hippocampal neurons [57,58]. Mitochondrial dysfunction contributes to aging-related alterations [14,59]. In the brain, there are at least two mitochondrial populations; which differ according to their origin [24,60]. Non-synaptic mitochondria originate from glial and neuronal cells; meanwhile, synaptic mitochondria are obtained exclusively from synaptic regions of the neuron (synaptosomes) [61]. Here, we evaluated the bioenergetics function and the calcium buffering capacity of synaptic and non-synaptic mitochondria from the hippocampus. We dissected the hippocampus of 3, 6, 12, and 18mo mice, and we isolated the synaptic (contained in synaptosomes) and non-synaptic mitochondria using a Percoll Gradient (Fig. 3A) [28]. We measured the bioenergetic function of the ETC of both mitochondrial populations, by measuring: i) ROS and ii) ATP production, 30 min after the addition of oxidative substrates [48,49]. Interestingly, when we evaluated ROS production after exposure to pyruvate-malate substrates in the synaptic mitochondria, we observed that 6mo mice showed a tendency to increase the amount of ROS compared to 3mo mice, an effect that is significant at 12mo; whereas 18mo mice did not present differences with 3mo (Fig. 3B). In contrast, non-synaptic mitochondria did not present significant differences in ROS production between all groups (Fig. 3C). To demonstrate whether these changes in ROS result in defects in ATP production, we evaluated ATP concentration in the medium of synaptic and non-synaptic mitochondria after exposure to pyruvate-malate substrates, using a bioluminescent assay. Surprisingly, we observed that the synaptic mitochondria obtained from 12 and 18mo mice had a significantly lower ATP production rate compared with 3mo animals (Fig. 3D); meanwhile, non-synaptic mitochondria only presented a significant reduction in 18mo animals (Fig. 3E). Altogether, these results indicate that both synaptic and non-synaptic mitochondria from the hippocampus reduce their bioenergetics function with age, but synaptic mitochondria fail prematurely at 12mo, generating an increase in ROS production and a deficit in ATP formation.

Finally, we evaluated the response of synaptic and non-synaptic mitochondria to calcium overload (Fig. 3F and G). For this, we measured absorbance at 540 nm, where a decrease in the absorbance indicates mitochondrial swelling [50]. We measured the basal absorbance for 3 min; next, we added 20 μM of CaCl_2_ to the mitochondrial fractions and continued measurement until 15 min, finally we added 200 μM of CaCl_2_ and evaluated the response for another 15 min. Our results showed that synaptic mitochondria of the aged 18mo animals responded immediately to 20 μM calcium overload (Fig. 3F), suggesting that synaptic mitochondria from the hippocampus of 18mo are more prone to swelling when exposed to calcium, probably due to a rapid and permanent opening of mPTP [62]. Additionally, we observed that synaptic mitochondria from the hippocampus of 12mo mice showed a reduction in absorbance when 200 μM of CaCl_2_ was added; however, this decrease was less severe than the change observed at 18mo (Fig. 3F). This last result suggests that synaptic mitochondria are more sensitive to calcium overload from 12mo onwards. In contrast, when evaluating the non-synaptic mitochondrial response to calcium, we observed that only mitochondria from 18mo showed a slight reduction in absorbance when 200 μM of CaCl_2_ was added (Fig. 3G), similar to that observed in 12mo mice in synaptic mitochondria. These results indicate that synaptic mitochondria from the hippocampus are more susceptible to calcium overload than non-synaptic mitochondria, resulting in premature swelling from 12mo and onwards at lower calcium concentrations.

Together our results demonstrate that synaptic mitochondria from the hippocampus present bioenergetic and calcium-regulatory defects of premature manner compared with non-synaptic mitochondria, presenting alterations since 12 month-old. Considering the importance of correct calcium buffering in the pre-synaptic region and optimal ATP concentrations to supply the synaptic demand, is possible that this synaptic mitochondrial dysfunction may contribute to the memory loss described previously.

### 3.4 MitoQ or Curcumin (C*c*) treatment prevents synaptic mitochondrial dysfunction during aging

**Fig. 4:**
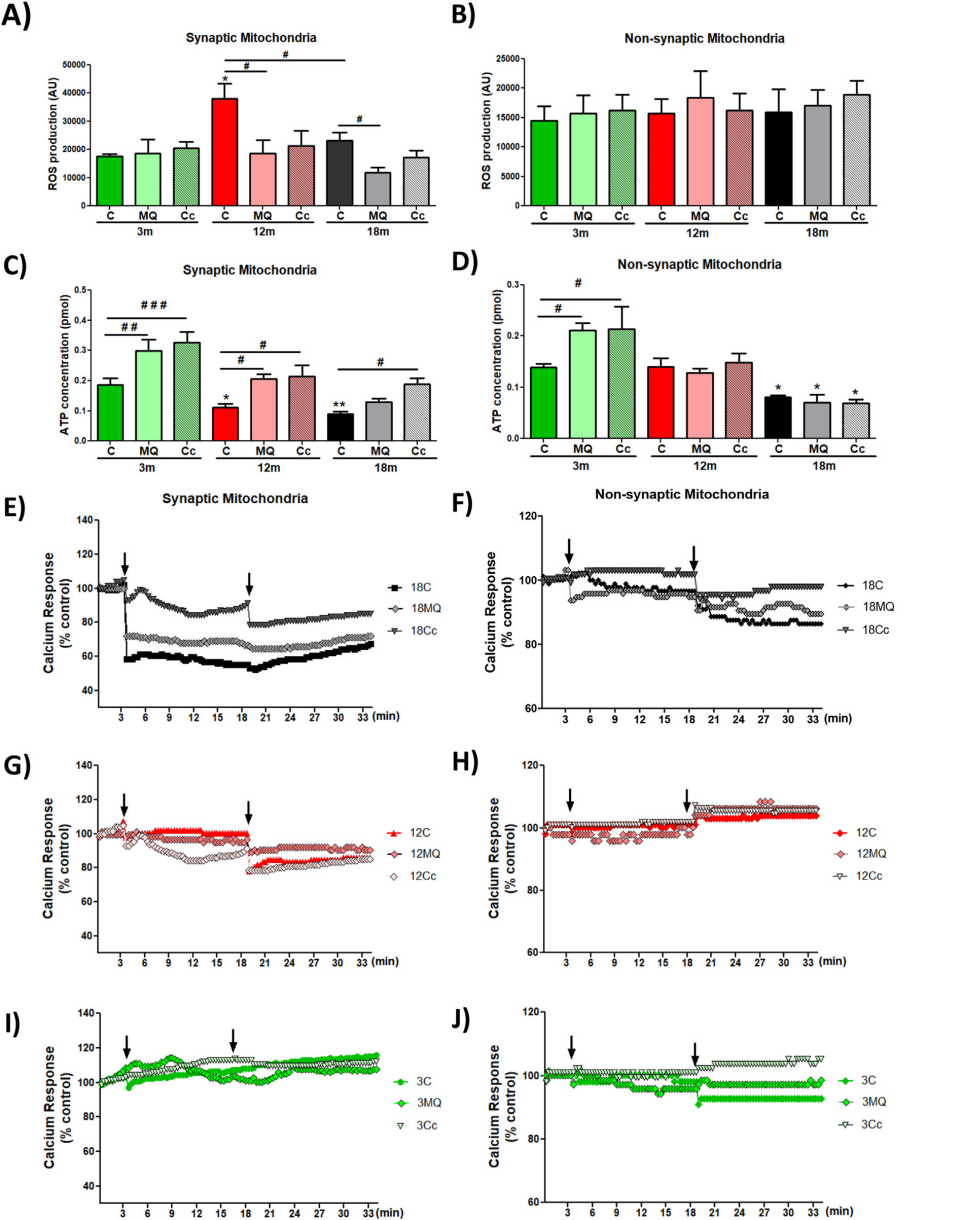
**Treatment with mitochondria-targeted antioxidant MitoQ and Curcumin prevents the loss of synaptic mitochondria in 12 and 18 month-old mice**. ROS production by (A) synaptic and (B) non-synaptic mitochondria, 30 min after exposure to oxidative substrates. ATP concentration produced by (C) synaptic and (D) non-synaptic mitochondria. Response to calcium overload by (E, G, I) synaptic and (F, H and J) non-synaptic mitochondria of 18, 12 and 3 month-old mice respectively; after exposure to 20 μM and 200 μM CaCl_2_. Graph bars represent means ± SEM. * indicate significant differences (p < 0.05) with 3mo control group. # indicate significant differences between control and treated-mice of the same age. #p < 0.05. ##p < 0.01; ###p < 0.001.

Mitochondrial dysfunction is considered a hallmark of aging because its aggravation contributes to the aging phenotype [21]. For this reason, diverse anti-aging treatments target mitochondria, mainly decreasing oxidative damage [31,63]. Therefore, we used MitoQ and *Cc* to evaluate if synaptic mitochondrial dysfunction could be prevented. Mice were exposed to treatment with i) MitoQ (250 μM in water, ad libitum) or Curcumin (i.p. injection) for 5 weeks. After treatment, we isolated non-synaptic and synaptosomal fractions containing synaptic mitochondria. We measured the production of ROS and ATP in synaptic and non-synaptic mitochondria of the hippocampus to see if treatment prevents mitochondrial dysfunction during aging. First, we evaluated ROS production after the addition of oxidative substrates. In synaptic mitochondria, we observed that 12mo mice showed increased ROS production, an effect that was attenuated by MitoQ, and a similar tendency was observed in the Curcumin-treated group (Fig. 4A). Likewise, ROS production was decreased in 18mo mice with MitoQ treatment, a tendency that was also observed in the Curcumin-treated synaptic mitochondria (Fig. 4A). In contrast, non-synaptic mitochondria presented no significant differences in ROS production after MitoQ and Curcumin treatment (Fig. 4B), indicating that the antioxidant properties of both treatments are specific to synaptic mitochondria.

Subsequently, we evaluated ATP production in synaptic mitochondria of the hippocampus. Interestingly, we observed that at 3mo MitoQ and Curcumin treatment significantly increased ATP production compared to the control group; similar to 12mo mice where both treatments increased ATP concentration (Fig. 4C). Finally, at 18mo MitoQ and Curcumin group showed an increase in ATP production, an effect that only is significant with Curcumin compared to 18mo control group (Fig. 4C). Lastly, we determined ATP production in the non-synaptic mitochondria of the hippocampus after treatment, observing that only the 3mo MitoQ group significantly increased its ATP production compared to the 3mo control group (Fig. 4D). In any other age, treatments modified the ATP concentration (Fig. 4D), suggesting that both treatments improve the bioenergetic function of synaptic mitochondria of the hippocampus, reducing premature dysfunction at the synapses.

On another hand, we evaluated the response of synaptic and non-synaptic mitochondria to calcium overload after treatment with MitoQ and Curcumin. Our results showed that Curcumin treatment, in contrast to MitoQ, prevents the mitochondrial swelling in response to 20 μM and 200 μM CaCl2 in both synaptic (Fig. 4E) and non-synaptic mitochondria (Fig. 4F) at 18mo. In addition, in the 12mo groups, we observed that MitoQ and Curcumin tend slightly to prevent the mitochondrial swelling of synaptic mitochondria after 20 μM and 200 μM CaCl2 exposure (Fig. 4G). In contrast, both treatments showed no significant differences in non-synaptic mitochondria from 12mo (Fig. 4H); and 3mo (Fig. 4I and J). These results suggest that both MitoQ and Curcumin treatments prevent mitochondrial swelling previously observed since 12mo, reducing premature dysfunction of synaptic mitochondria.

### 3.5 Treatment with MitoQ or Curcumin prevents recognition memory loss in 12-month-old mice

**Fig. 5:**
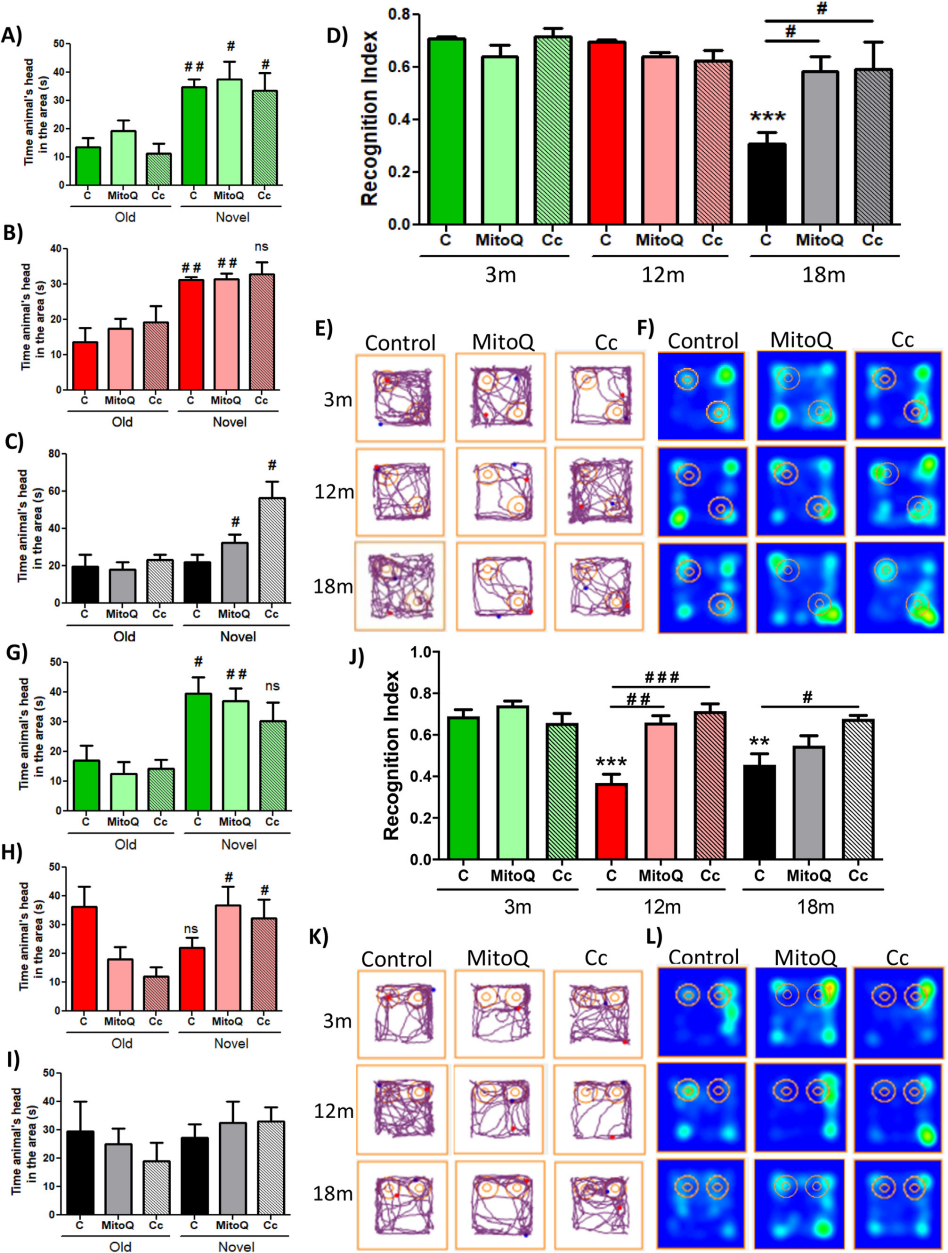
**MitoQ and Curcumin treatment prevent recognition memory impairment from 12 month-old and onwards**. Time that the animals spent exploring old and novel object localization at (A) 3month-old, (B) 12month-old and (C) 18month-old. (D) Recognition Index of each group in NOL test. (E) Representative track of one animal per group during the NOL test. (F) Heat maps representing the behavior of each group in the NOL test. The time that the animals of (G) 3month-old, (H) 12month-old and (I) 18month-old, spent exploring old and novel objects. (J) Recognition Index of each group in NOR test. (K) Representative track of one animal per group during NOR tests. (L) Heat maps representing the behavior of each group in the NOR test. Graph bars represent means ± SEM. * indicate significant differences with 3mo control group. *p < 0.05. **p < 0.01; ***p < 0.001. # indicate significant differences between control and treated-mice of the same age. #p < 0.05. ##p < 0.01; ###p < 0.001.

Since mitochondrial function is key for correct synaptic communication [8,64], we determined if the improvement of synaptic mitochondrial activity could prevent memory impairment observed with age. We used the NOL test to evaluate object localization memory in control and treated groups. We observed that 3mo control- and treated-mice had similar behavioral preferentially explored the novel object (Fig. 5A). Similarly, all groups of 12mo mice spent more time exploring the novel object (Fig. 5B). In contrast, only the 18mo MitoQ- and Curcumin-treated group showed significant differences compared to the control group, reverting the loss of object localization memory showed at 18mo (Fig. 5C). Recognition Index is shown in Fig. 5D, where MitoQ and Curcumin treatment showed differences from the control group at 18mo. The behavior of a representative animal per group is shown in the tracks of Fig. 5E; and these differences are more clearly shown in the heat map of each group, where MitoQ and Curcumin treated 12 and 18mo mice spent more time in the novel localization (Fig. 5F). Thus, these results indicate that both MitoQ and Curcumin treatment reduce or prevent object localization memory loss.

Next, we performed the NOR task and our results revealed that the 3mo control, MitoQ, and Curcumin groups spent more time exploring the novel object (Fig. 5G); although only control and MitoQ groups showed significant differences in the time exploring the old and the novel object, possibly by the variability between the animals in each group. Interestingly, we also observed that 12mo mice treated with MitoQ or Curcumin explored the new object for longer, in contrast to the 12mo control group (Fig. 5H), indicating that both treatments impede recognition memory impairment at this age. Finally, when we analyzed the behavior of 18mo experimental groups, we observed a tendency towards a preference for the novel object in both MitoQ and Curcumin animals (Fig. 5I). In fact, the Recognition Index showed significant differences in the 12mo MitoQ- and Curcumin-treated mice, as well as between 18mo control and Curcumin-treated mice (Fig. 5J). A representative track of each group is shown in Fig. 5K; likewise, Fig. 5L shows the heat map summarizing the behavior of the group. These results indicate that treatments with MitoQ and Curcumin have a positive effect on aging, attenuating object recognition memory loss in 12 and 18mo mice. All these observations strongly suggested that preserving the function of synaptic mitochondria could prevent object recognition and localization memory loss during aging.

### 3.6 Treatment with MitoQ or Curcumin improves spatial memory in 18 month-old mice

**Fig. 6:**
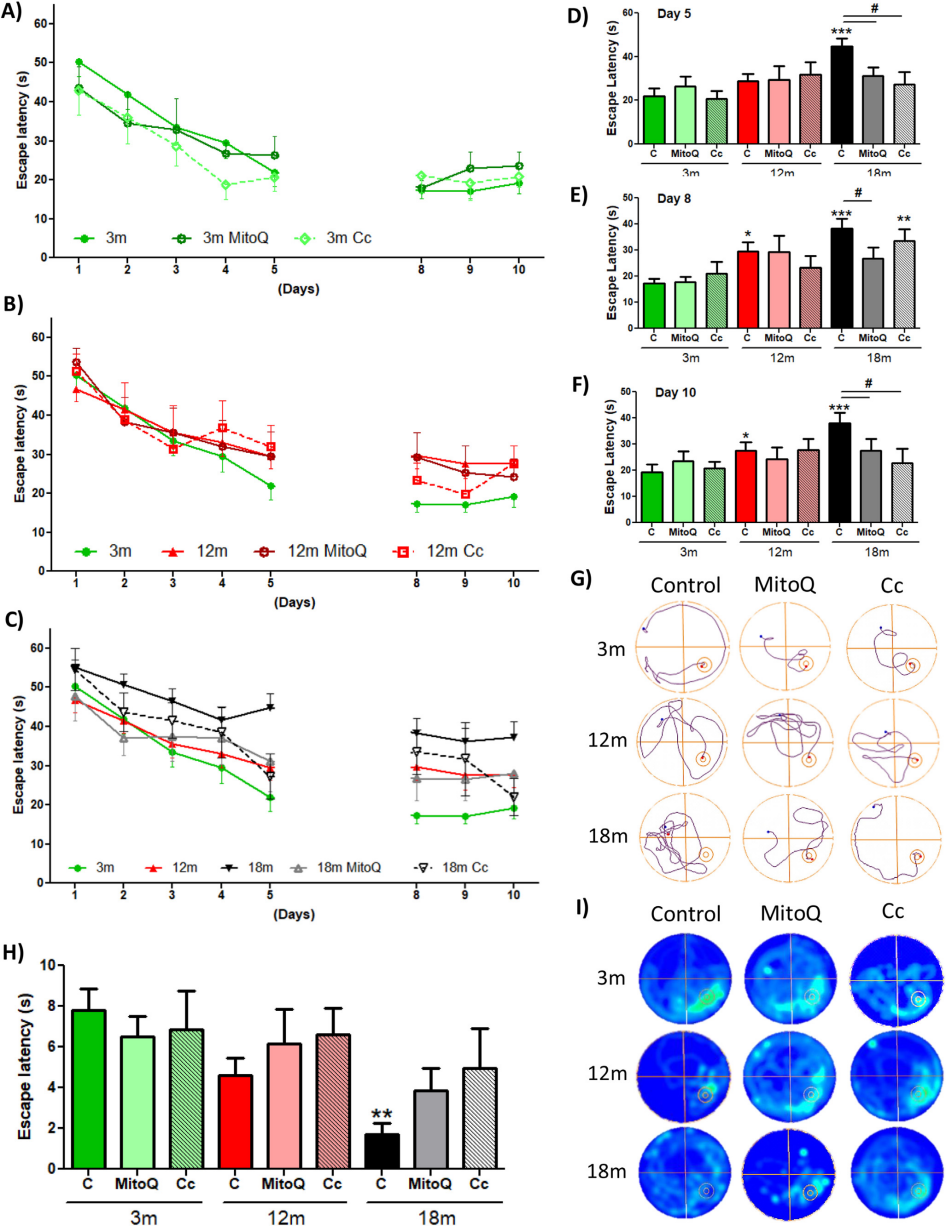
**Improvement of spatial memory in 18 month-old mice after treatment with MitoQ and Curcumin**. Escape latency of (A) 3 month-old, (B) 12 month-old and (C) 18month-old mice during the MWM test after treatment. Graph indicating significant differences at: (D) 5th day, (E) 8th day and (F) 10th day of training after treatment. (G) Representative track of one animal per group after treatment. (H) Time in the area of the platform during the Probe test. (I) Heat maps represent the behavior of each group in the Probe test. Graph bars represent means ± SEM. * indicate significant differences with 3mo control group. *p < 0.05. **p < 0.01; ***p < 0.001. # indicate significant differences between control and treated-mice of the same age. #p < 0.05.

To determine whether MitoQ and Curcumin treatment modify spatial memory impairment in 18mo mice, we performed the MWM test. Considering that previously the most important differences were observed in the MWM test, we decided to use only this test after the treatments. We observed that in the 3mo group, treatment with MitoQ and Curcumin did not show significant differences in the escape latency compared to the control group (Fig. 6A). The 12mo control, MitoQ, and Curcumin groups presented similar escape latencies between them and with the 3mo control mice during the first five days of training (Fig. 6B and D). However, during the 8th and 10th day, a significant difference between control animals at 3mo and 12mo was observed (Fig. 6B, E, and 6F); in contrast to 12mo MitoQ and Curcumin-treated mice that did not present differences with 3mo control mice (Fig. 6B, E and 6F). Finally, we analyzed the behavior of 18mo groups. We observed that 18mo control mice presented a higher escape latency compared to 18mo MitoQ- and Curcumin-treated mice (Fig. 6C–F), suggesting that both 18 m treated groups were quicker at learning the localization of the hidden platform than the 18mo control group (Fig. 6C and D). Likewise, in the second week of training, we observed a significant difference between the 18mo animals, where MitoQ treatment improved memory regarding the position of the platform on day 8 (Fig. 6C and E) and both 18mo treated-groups presented higher spatial memory at 10th day (Fig. 6C and F). The track of a representative mouse of each group on day 10 is shown in Fig. 6G. Subsequently, we performed the Probe test (11th day). We observed that the control group of 18mo mice spent significantly less time in the platform area, unlike MitoQ and Curcumin groups of the same age, which presented similar escape latency to the 12mo groups (Fig. 6H). Fig. 6I showed the heat map of each group in the probe test. Here it is possible to observe the differences between the behavior of 18mo groups. These results indicate that after treatment of 12mo and 18mo mice there is a considerable improvement in both learning and spatial memory. Altogether, our results indicate that MitoQ and Curcumin can prevent the loss of cognitive functions, including spatial memory.

### 3.7 Treatment with MitoQ or Curcumin improves mitochondrial structure in 12 and 18 month-old mice

**Fig. 7:**
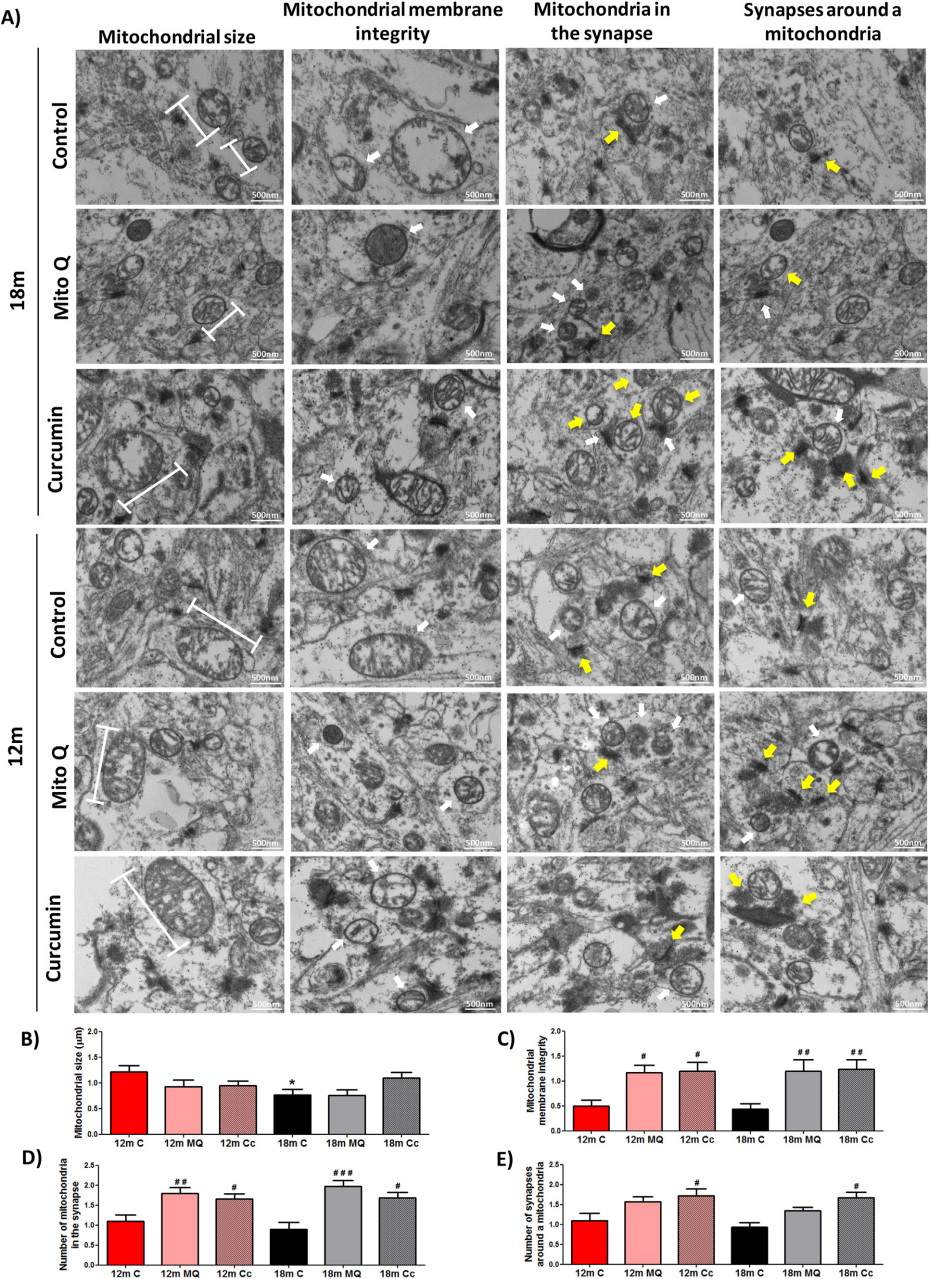
**MitoQ and Curcumin treatment prevents mitochondrial structural damage and strengthen the synapse in 12 and 18 months-old mice**. (A) Electron microscopy of CA1 hippocampal synaptic mitochondria from 12 and 18 month-old mice. Graph indicates (B) size and (C) mitochondrial membrane integrity of synaptic mitochondria; (D) the number of mitochondria in one synapse; and (E) number of synapses around mitochondria of 12 and 18mo groups after MitoQ or Curcumin treatment. White arrows indicate synaptic mitochondria and yellow arrows indicate a synapse. *Significant differences with 3mo control group. Graph bars represent means ± SEM. * indicate significant differences (p < 0.05) with 3mo control group. # indicate significant differences between control and treated-mice of the same age. #p < 0.05. ##p < 0.01. (For interpretation of the references to colour in this figure legend, the reader is referred to the Web version of this article.)

Mitochondria are dynamic organelles exhibiting changes in their size and morphology, which are closely associated to its functionality [65]. Previously, we showed that MitoQ and Curcumin treatments improve the function of hippocampal synaptic mitochondria from 12 and 18mo mice. Therefore, we evaluated if these antioxidants also improve mitochondrial structure using transmission electron microscopy (Fig. 7). First, we studied the size of synaptic mitochondria from the CA1 hippocampus and we observed that the mitochondria of control 12mo mice present a larger size compared to 18mo control group (Fig. 7A and B). However, both MitoQ and Curcumin treatments showed no changes in the size of synaptic mitochondria from 12 and 18mo mice (Fig. 7A and B). Subsequently, we evaluated the mitochondrial membrane integrity (Fig. 7C), observing that 12 and 18mo MitoQ and Curcumin groups present a significant increment in the integrity of the mitochondrial membrane compared to 12 and 18mo control group respectively, which are visibly damaged (Fig. 7A and C). Then, we analyzed the number of mitochondria for each synapse (Fig. 7A and D). Surprisingly, we observed that 12mo MitoQ group present significantly more mitochondria around one synapse, compared to 12mo control group (Fig. 7A and D). More importantly, our results reveal that 18mo MitoQ and Curcumin groups presented more mitochondria in a synapse compared to 18mo control group (Fig. 7A and D). This recruitment of more mitochondria in the synapses is possibly to provide higher energy for synaptic communication. Finally, we studied the number of synapses around mitochondria (Fig. 7A and E). Interestingly, we observed that at 12mo only Curcumin group presented a significant increase in the number of synapses around mitochondria compared to control mice (Fig. 7A and E). Similarly, we observed that 18mo Curcumin group presented more synapses around mitochondria compared to 18mo control group (Fig. 7A and E); suggesting that Curcumin treatment also contributes to the generation of new synapses near to mitochondria. We also evaluated these parameters in all groups of 3mo, but no significant differences were observed (Supplementary Figure 4). These results indicate that treatment with MitoQ or Curcumin not only improves synaptic mitochondrial function in 12 and 18mo mice but also improves the structure of the mitochondria and the synapses; possibly enhancing hippocampal memory.

### 3.8 Decreased ATP production of synaptic mitochondria correlates with behavioral impairment during aging

**Fig. 8:**
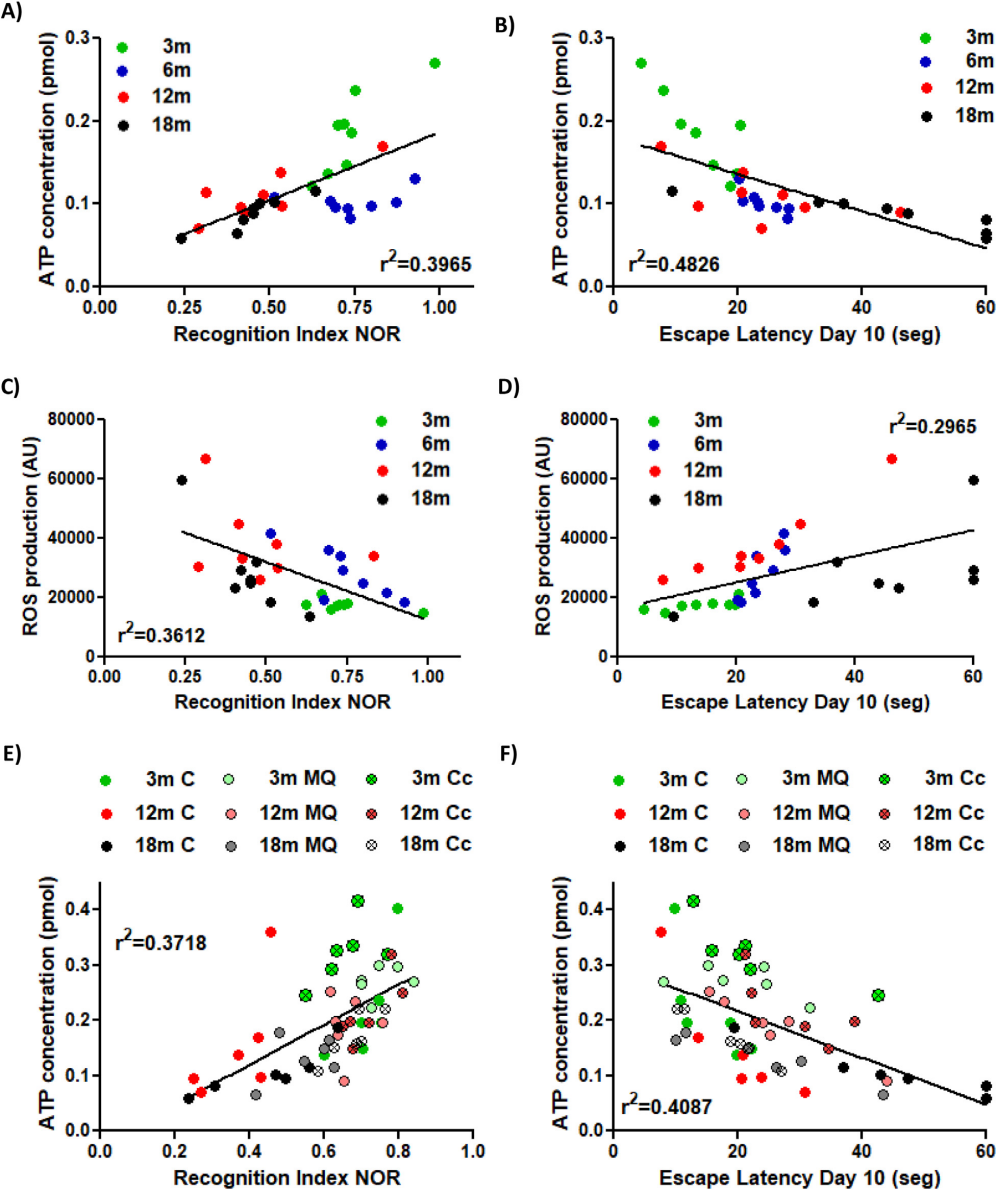
**Pearson's correlation analysis between synaptic mitochondrial bioenergetics function and cognitive impairment age-related**. Pearson's correlation analysis was performed to analyze the correlation between: ATP produced by synaptic mitochondria and (A) the Recognition Index of NOR test or (B) Escape Latency on day 10 of Morris Water Maze test of 3, 6, 12 and 18 month-old mice; ROS produced by synaptic mitochondria and (C) the Recognition Index of NOR test or (D) Escape Latency on day 10 of Morris Water Maze test of 3, 6, 12 and 18mo mice; ATP produced by synaptic mitochondria and (E) the Recognition Index of NOR test or (F) Escape Latency on day 10 of Morris Water Maze of 3, 12 and 18mo mice control, treated with MitoQ or Curcumin.

To investigate whether the bioenergetics function of synaptic mitochondria was associated with the impairment of recognition and spatial memory, we conducted a Pearson's correlation analysis between the ATP or ROS produced by synaptic mitochondria and behavioral indexes of 3, 6, 12 and 18mo mice (Fig. 8A–D). Fig. 8A shows that ATP production of synaptic mitochondria was positively associated with the Recognition Index of NOR test (r^2^ = 0.3965); whereas it was negatively correlated with the escape latency on day 10 of (MWM) test (r^2^ = 0.4826) (Fig. 8B). A similar correlation, but with a minor correlation index, was found between the levels of ROS produced by synaptic mitochondria and the Recognition Index of NOR test (r^2^ = 0.3612) (Fig. 8C) or the escape latency on day 10 of MWM test (r^2^ = 0.2965) (Fig. 8D). Thus, these results indicate that increased ATP production of synaptic mitochondria correlated with improved hippocampal cognitive performance, at the same time that increased ROS production of synaptic mitochondria correlates with cognitive impairment. Finally, to validate if the recovery of synaptic mitochondrial function induced by MitoQ or Curcumin treatment correlates with the cognitive improvement, Pearson's correlation analysis between ATP production of synaptic mitochondria and Recognition Index of NOR test (Fig. 8E) or the escape latency on day 10 of MWM test (Fig. 8F) of control and treated animals was performed. Interestingly, we also observed a correlation between ATP produced by synaptic mitochondria and the cognitive capacity of adult and aged mice, as indicated by an r^2^ = 0.3718 to ATP concentration and Recognition Index of NOR test and r^2^ = 0.4087 between ATP concentration and the escape latency on day 10 of MWM test (Fig. 8E and F). Therefore, these results reveal a correlation between the bioenergetics function of mitochondria of the synapses and the cognitive function of the hippocampus and propose that synaptic mitochondrial function is key to maintain the recognition and spatial memory.

In conclusion, our results demonstrate for the first time that synaptic mitochondria from the hippocampus fail before non-synaptic mitochondria during aging, demonstrating a premature mitochondrial dysfunction at the synapse. More importantly, we showed that the bioenergetics function of synaptic mitochondria correlated with cognitive performance, strongly suggesting that mitochondrial function at synapses contributes to the hippocampal memory formation. Thus, preventing the damage of synaptic mitochondrial structure and function is sufficient to attenuate cognitive alterations associated with hippocampal function such as memory.

## 4 Discussion

In the present study, we report hippocampus-dependent memory impairment during aging. Specifically, we demonstrated that recognition memory is initially observed at 12mo, whereas localization memory and spatial memory impairment occurred at 18mo. More importantly, we demonstrated a premature synaptic mitochondrial dysfunction, because this occurs before non-synaptic mitochondria, and is evidenced by increased ROS formation, decreased ATP production, and higher calcium sensitivity from 12mo of age. To demonstrate that synaptic mitochondrial dysfunction contributes to memory loss, we treated mice with the MitoQ or Curcumin for 5 weeks. Surprisingly, we reported that treatments were able to prevent synaptic mitochondrial defects, without affecting the non-synaptic mitochondria. More interestingly, we showed that restoring the structure and function of synaptic mitochondria is sufficient to attenuate the memory impairment during aging, due that ATP production by synaptic mitochondria correlates with the cognitive performance age-related. Therefore, our results indicate that dysfunction of the synaptic hippocampal mitochondria contributes to memory loss in aging.

Memory loss is common during aging [66]. The hippocampus is a crucial structure for recognition and spatial memory [67]. Here, we used a cognitive test to evaluate hippocampal-dependent memory, detecting that the most severe changes were observed in the NOR task. We report that 12mo mice present altered object recognition, whereas localization recognition and spatial memory loss were observed at 18mo. This is consistent with previous reports from C57BL/6 mice presenting alterations associated with hippocampal function with age [68,69], which are early observed in the recognition memory [70]. These hippocampal-related changes have also been reported in humans and in other animal models [56,71,72], indicating that a loss of hippocampal function is a common characteristic of aging, validating our results.

Oxidative stress is a cellular characteristic of the aged brain. In fact, it is one of the most studied hypotheses to explain the changes that occur at an advanced age [73]. During aging, there is an imbalance between oxidative molecules and antioxidant defense that result in increased ROS [74,75]. Astrocytes from the cerebral cortex and hippocampus of C57BL/6 mice showed that aged mice present significantly increased ROS production [76]. This same increased ROS production is involved in cardiac diseases [77] and neurodegenerative diseases [78]; suggesting that increased ROS production could contribute to neurological alterations associated with pathologies.

Mitochondria are the main ROS producers, as a sub-product of the ETC [79] that leads to ATP production [80]. In neurons, it is possible to find mitochondria in the neuronal soma, but also in the neurites that result in pre- and post-synaptic sites for synapses [81]. For this reason, the mitochondria in the brain can be classified into synaptic and non-synaptic mitochondria [30]. Diverse studies have proposed structural and functional differences between both mitochondrial pools in the whole brain [60] or the cerebral cortex [[28], [29], [30]]. Regarding the hippocampus, only one study proposes a differential response between mitochondrial populations in hypothyroid conditions [82]. In aging, studies have shown that synaptic and non-synaptic mitochondria from the cerebral cortex present different functionalities, where synaptic mitochondria are damaged before the non-synaptic population [29,30,83]. However, this has not been explored in the hippocampus of aged mice. Here, we showed that synaptic mitochondria from the hippocampus of 12mo mice presented a premature bioenergetic dysfunction, evidenced by increased ROS production and reduced ATP formation; an effect that does not occur in the non-synaptic population at 12mo. Additionally, we observed reduced ATP formation in both synaptic and non-synaptic mitochondria at 18mo. This differential function observed between synaptic and non-synaptic mitochondria from the hippocampus could be due to changes in the expression or activity of the ETC complexes [84], accompanied by alterations in the antioxidant enzymes during aging [85]. This could also be due to the different evolution of synaptic transmission against neuronal activity or to the change induced by the alteration of multiple neuromodulation systems in the aging process [86]. Future studies could help address this question.

Another important function of the mitochondria is to regulate calcium concentrations [87]. The mPTP regulates calcium homeostasis, and for this process, this pore is transiently open [88]. Nevertheless, against high calcium concentrations, the mitochondria are incapable of regulating its concentrations, leading to a permanent mPTP opening, which results in mitochondrial swelling and finally apoptosis [88]. Studies performed in extracted synaptic and non-synaptic mitochondria from the cortex indicate that the mitochondria localized in the synapses are more susceptible to damage by constant calcium changes [28]. These differences seem to increase during aging, as shown by a negative response of synaptic mitochondria after calcium-induced depolarization [29]. Regarding the hippocampus, to date, there are no studies that show if these differences also occur in this brain region. Here, we reported calcium buffering dysfunction during aging, an effect that is more drastic and premature in synaptic mitochondria. This last factor is demonstrated by mitochondrial swelling with high calcium concentrations at 12mo; an effect that does not occur in the non-synaptic population at the same age. Also, we observed a severe sensibility to calcium overload in synaptic mitochondria at 18mo, whereas hippocampal non-synaptic mitochondria from 18mo mice only presented a lower sensibility, similar to synaptic mitochondria at 12mo of age. This could be explained by a deterioration of the cell calcium homeostatic mechanisms towards increased intracellular [Ca^2+^] in old age [89,90]. Another possibility is the increased activity of mPTP, possibly due to increased expression of proteins involved in mPTP formation as cyclophilin D (Cyp-D), which promotes its opening [91]. In fact, in the cerebral cortex a study demonstrated that synaptic mitochondria possess high levels of Cyp-D compared to non-synaptic mitochondria, resulting in increased swelling by the mPTP opening [92]. Additional studies could validate whether this also occurs in mitochondrial population of the hippocampus.

Adequate function of synaptic mitochondria is fundamental to synapses and therefore to the processes of synaptic plasticity that promotes memory formation, such as exocytosis of vesicles containing neurotransmitters [93]; spinogenesis [94]; or long-term potentiation (LTP) and long-term depression (LTD) [8,95]. In fact; eliminating mitochondria from dendrites result in a loss of synapses and dendritic spines; whereas this effect is recovered by an accumulation of mitochondria in the dendrites [94]. Also, mitochondrial fission in the dendritic spines in necessary to carry out LTP [96]. Finally, studies have shown that structural alterations of synaptic mitochondria correlated with impaired working memory [97]; whereas improving mitochondrial function significantly attenuated the cognitive decline in aging [98].

Our work corroborates these earlier findings by showing that the dysfunction of synaptic mitochondria of the hippocampus contributes to the memory loss observed in aging. This last is showed by Pearson's correlation analysis, which demonstrates a positive correlation between the ATP concentration produced by synaptic mitochondria and the hippocampus-dependent cognitive capacities. For this, we exposed mice to treatment with MitoQ or Curcumin. Studies have been conducted in which MitoQ offers protective and favorable effects against neurodegenerative diseases [99]; proposing that this could have a future pharmacological use considering that mitochondrial dysfunction is key to many diseases [100]. Another studied antioxidant is Curcumin, described as a protector against the lesions induced by oxidative stress in neurodegenerative diseases [101]. In our results after treatment, we observed that both MitoQ and Curcumin significantly improved synaptic mitochondrial structure and function. MitoQ significantly reduces the oxidative stress observed in elderly animals; restoring the mitochondrial membrane integrity and recruit more mitochondria to unique synapse. While Curcumin had a greater effect on ATP production in synaptic mitochondria of the hippocampus; preventing mitochondrial swelling at 18mo and promoting the formation of multiple synapses around mitochondria. With these findings, we suggested that Curcumin could act as a neuroprotector, like MitoQ, and also, it could be directly related to the improvement of mitochondrial function. Simultaneously, we observed that by improving synaptic mitochondrial function we attenuated or prevented the loss of recognition and spatial memory during aging. Thus, we demonstrated that maintaining the function of synaptic mitochondria could prevent hippocampus-dependent cognitive alterations at an advanced age. Nowadays it is known that MitoQ is an antioxidant that acts directly on the mitochondria, favoring functions in such a way that it diminishes mitochondrial oxidative damage [102]. Since Curcumin had similar results to MitoQ, we also could indicate that they act as an antioxidant, but also could play a role in regulating calcium levels and modulating the synapse. To date, diverse action targets have been proposed to Curcumin [43], including an antioxidant effect [103]; increasing Cu/Zn SOD and PARP-1 activity [104], increasing activities of antioxidant enzymes [105] and acting as a potent anti-inflammatory [43]. Future studies could determine if Curcumin acts directly on the mitochondria, preventing their dysfunction.

In conclusion, we demonstrated for the first time that synaptic mitochondria of the hippocampus fail before non-synaptic mitochondria, which results in impairments in recognition and spatial memory. This proposes that the age-related cognitive impairment could be a consequence, almost in part, of the premature dysfunction of mitochondria at the hippocampal synapses. Also, we reported that preventing the dysfunction of synaptic mitochondria could be a new target for treating or impeding age-associated cognitive damage.

## Declaration of competing interest

The authors declare no conflict of interest.

## Appendix A Supplementary data

Supplementary Figure 1. Schematic representation of all cognitive tests. (A) Novel object localization (NOL), (B) Novel object recognition, (C) Barnes Maze (BM) and (D) Morris Water Maze (MWM).

Supplementary Figure 2. Representation of MitoQ or Curcumin treatment. 5 weeks of treatment with (A) MitoQ and (B) Curcumin in 3, 12 and 18 month-old mice.

Supplementary Figure 3. Water consumption of control and MitoQ treated mice. Water consumption of (A) 3, (B) 12 and (C) 18 month-old mice during the treatment.

Supplementary Figure 4. Treatment does not modify the mitochondrial structure at 3 month-old. (A) Electron microscopy of mitochondria from 3mo mice after treatment. Graph indicates (B) size and (C) mitochondrial membrane integrity of synaptic mitochondria; (D) the number of mitochondria in one synapse; and (E) the number of synapses around mitochondria.
