# Supplementary material for: Premature synaptic mitochondrial dysfunction in the hippocampus during aging contributes to memory loss
Source: Redox Biol. 2020 May 5;34:101558. doi: 10.1016/j.redox.2020.101558 (PMC7248293; doi:10.1016/j.redox.2020.101558)
Supplement: Multimedia component 1 [file mmc1.pptx]

## Slide 1
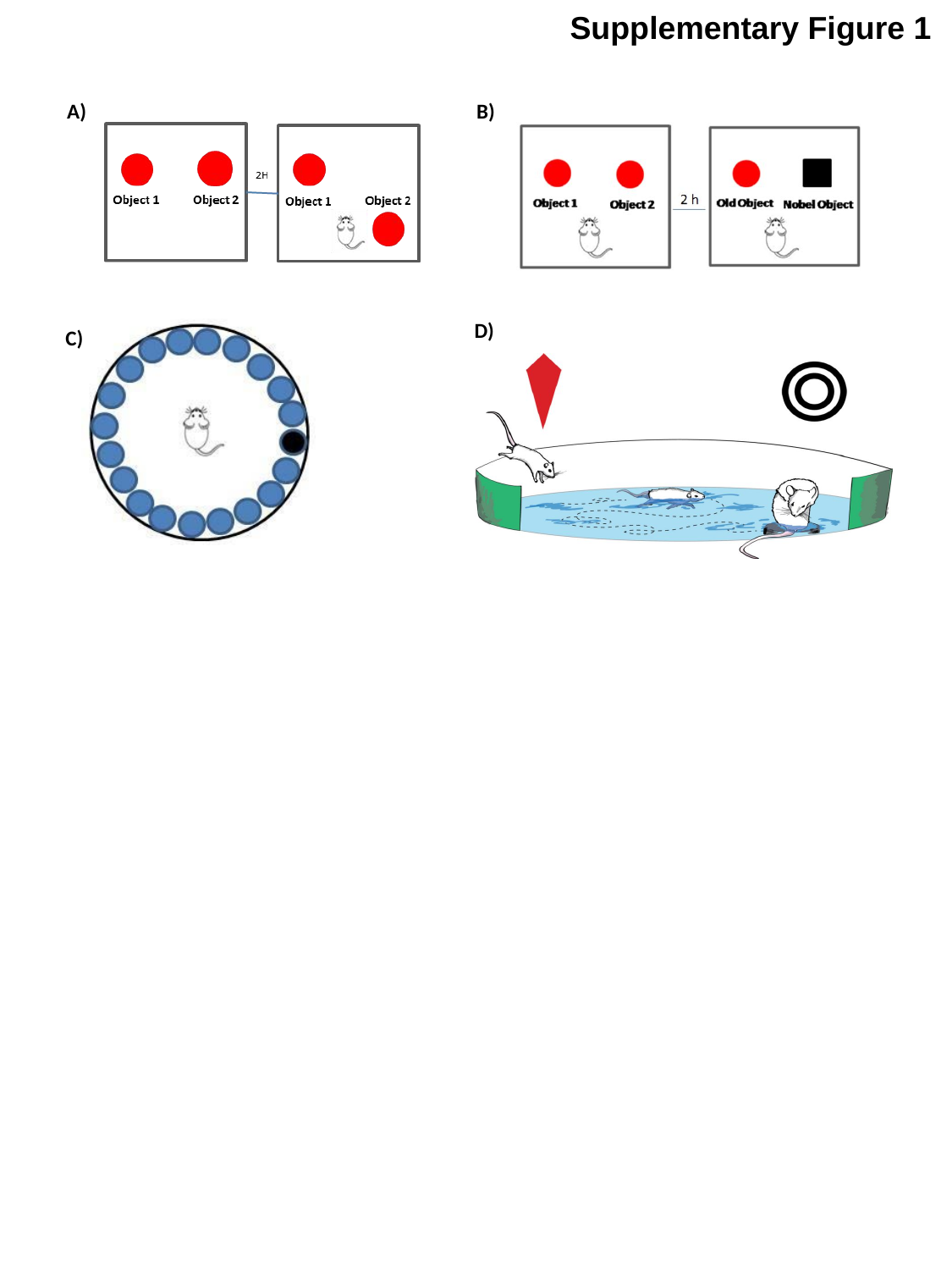

Supplementary Figure 1
A)
B)
D)
C)

## Slide 2
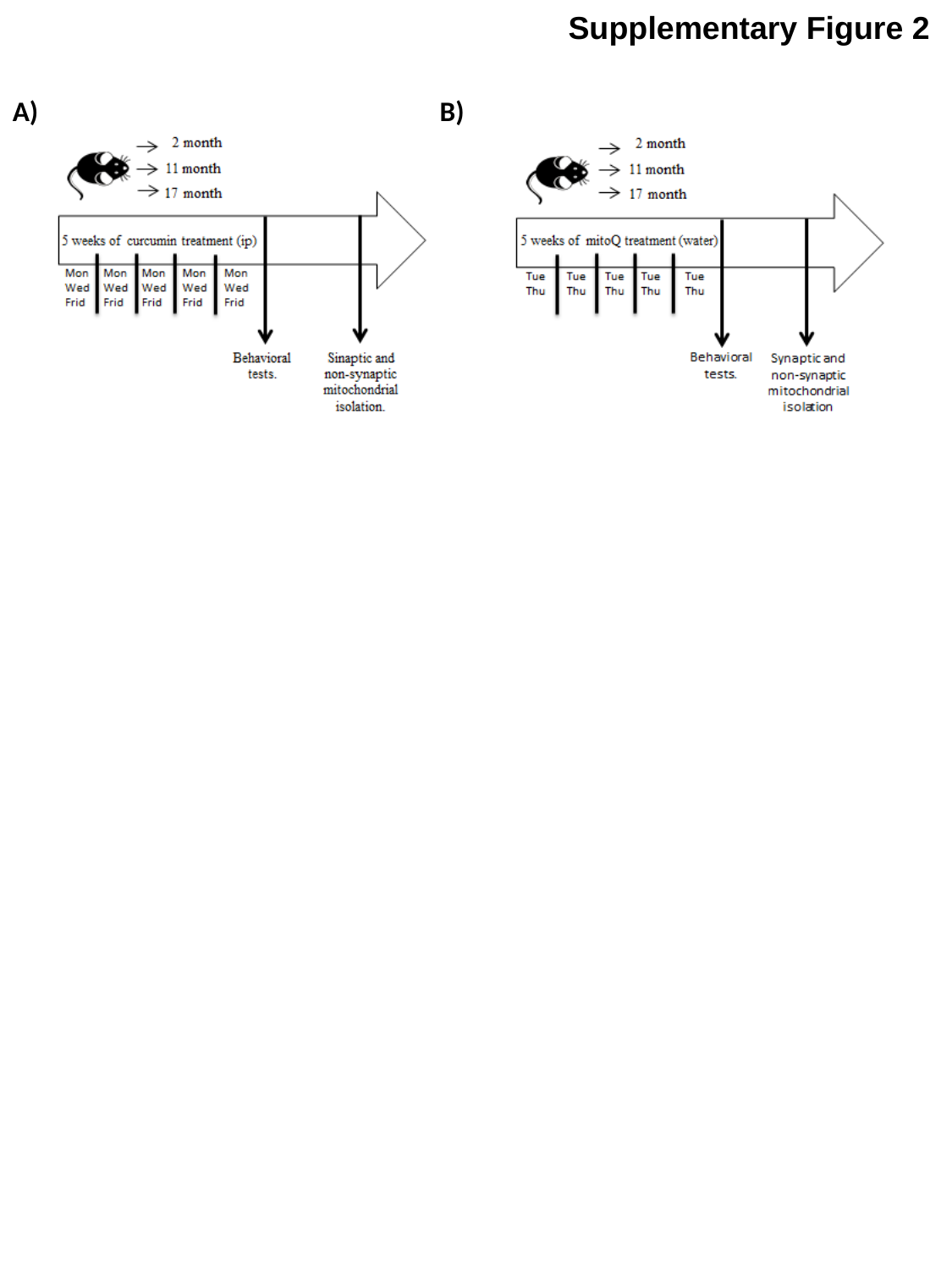

Supplementary Figure 2
A)
B)

## Slide 3
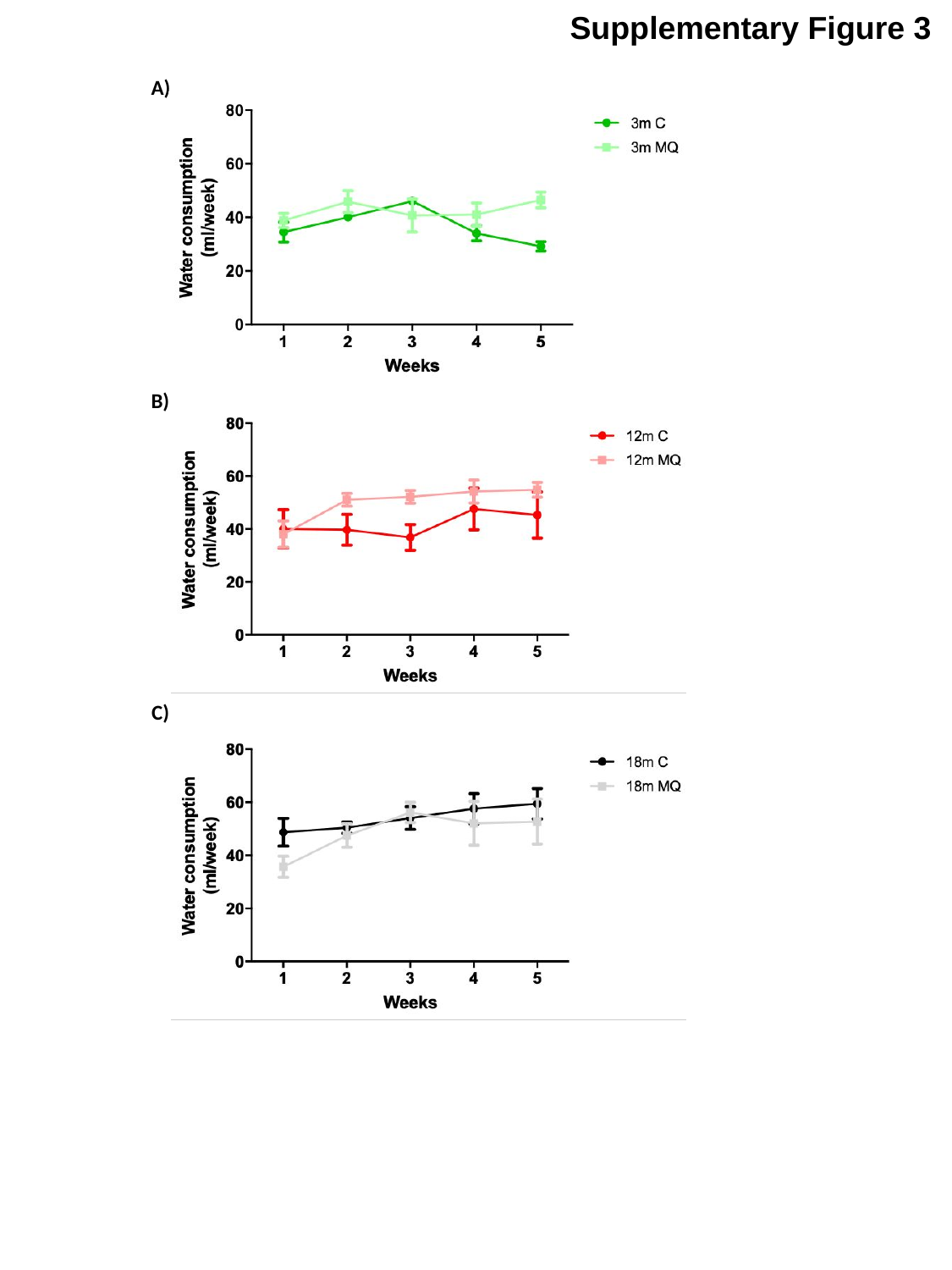

Supplementary Figure 3
A)
B)
C)

## Slide 4
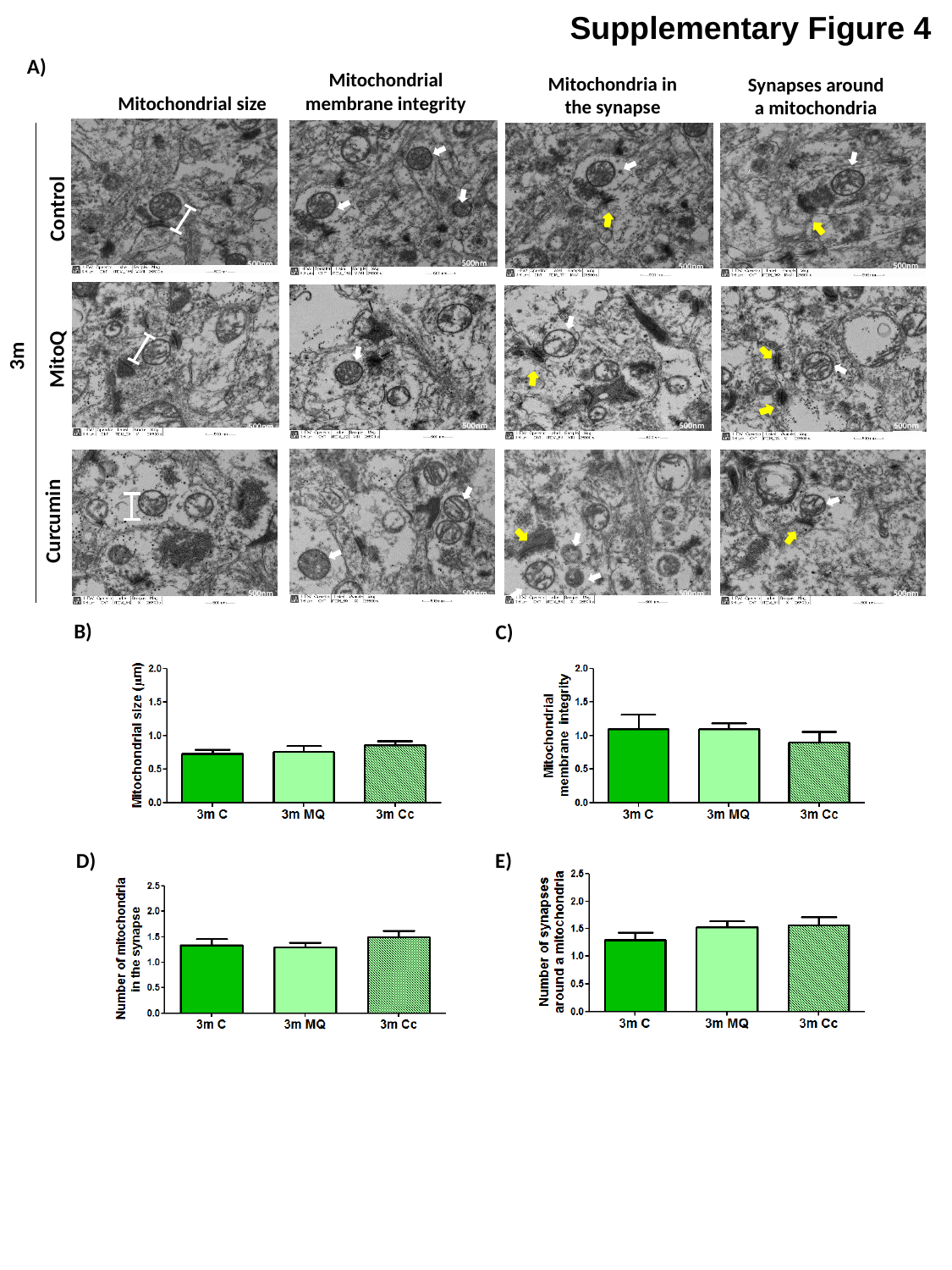

Supplementary Figure 4
A)
Mitochondrial membrane integrity
Mitochondria in the synapse
Synapses around a mitochondria
Mitochondrial size
Control
500nm
500nm
500nm
500nm
3m
MitoQ
500nm
500nm
500nm
500nm
Curcumin
500nm
500nm
500nm
500nm
B)
C)
E)
D)
